# Identification and Analysis of the Geohazards Located in an Alpine Valley Based on Multi-Source Remote Sensing Data

**DOI:** 10.3390/s24134057

**Published:** 2024-06-21

**Authors:** Yonglin Yang, Zhifang Zhao, Dingyi Zhou, Zhibin Lai, Kangtai Chang, Tao Fu, Lei Niu

**Affiliations:** 1 Institute of International Rivers and Eco-Security, Yunnan University, Kunming 650500, China; yang280082@163.com (Y.Y.);; 2 Kunming Survey, Design and Research Institute Co., Ltd., China Railway Eryuan Engineering Group Co., Ltd. (CREEC), Kunming 650200, China; 3 School of Earth Sciences, Yunnan University, Kunming 650500, China; 4 Engineering Research Center of Domestic High-Resolution Satellite Remote Sensing Geology for Universities of Yunnan Province, Kunming 650500, China; 5 Yunnan International Joint Laboratory of China-Laos-Bangladesh-Myanmar Natural Resources Remote Sensing Monitoring, Kunming 650051, China; 6 Yunnan Institute of Geological Science, Kunming 650051, China

**Keywords:** multi-source remote sensing, alpine valley, cascading geohazard, LiDAR, SBAS-InSAR

## Abstract

Geohazards that have developed in densely vegetated alpine gorges exhibit characteristics such as remote occurrence, high concealment, and cascading effects. Utilizing a single remote sensing datum for their identification has limitations, while utilizing multiple remote sensing data obtained based on different sensors can allow comprehensive and accurate identification of geohazards in such areas. This study takes the Latudi River valley, a tributary of the Nujiang River in the Hengduan Mountains, as the research area, and comprehensively uses three techniques of remote sensing: unmanned aerial vehicle (UAV) Light Detection and Ranging (LiDAR), Small Baseline Subset interferometric synthetic aperture radar (SBAS-InSAR), and UAV optical remote sensing. These techniques are applied to comprehensively identify and analyze landslides, rockfalls, and debris flows in the valley. The results show that a total of 32 geohazards were identified, including 18 landslides, 8 rockfalls, and 6 debris flows. These hazards are distributed along the banks of the Latudi River, significantly influenced by rainfall and distribution of water systems, with deformation variables fluctuating with rainfall. The three types of geohazards cause cascading disasters, and exhibit different characteristics in the 0.5 m resolution hillshade map extracted from LiDAR data. UAV LiDAR has advantages in densely vegetated alpine gorges: after the selection of suitable filtering algorithms and parameters of the point cloud, it can obtain detailed terrain and geomorphological information on geohazards. The different remote sensing technologies used in this study can mutually confirm and complement each other, enhancing the capability to identify geohazards and their associated hazard cascades in densely vegetated alpine gorges, thereby providing valuable references for government departments in disaster prevention and reduction work.

## 1. Introduction

Identification of potential and highly concealed geohazards and cascading geohazards is a hot topic in current geohazard research. Compared to single geohazards, cascading geohazards have higher concealment, greater risks, and difficulties in governance [1]. Situated in the collision zone between the Eurasian Plate and the Indian Ocean Plate, the Hengduan and the Himalayan Mountains possess a complex geological environment with active geological movements, resulting in numerous types of geohazards and cascading geohazards. Among them, cascading processes triggered by landslides and rockfalls are the most typical in the region [2,3,4], such as the “landslide-debris flow-river blockage” cascade in Sindupalchowk, Nepal, in 2014 [5]; the “landslide-debris flow-dammed lake-breach flood” cascade in Tibet, China, in 2018 [6]; and the “earthquake-landslide/rockfall-dammed lake-flood” cascade in Kukrekhola, Nepal, in 2018 [7]. In the Latudi River valley, a tributary of the Nujiang River in the Hengduan Mountains, the geological environment is fragile. Furthermore, the complex and continuous geological activities have fostered typical alpine gorge landforms. Influenced by both the Indian Ocean monsoons and the Pacific monsoons, there are two rainy seasons with concentrated precipitation. The frequent occurrence of geohazards in the area, with the county seat of Fugong located at the mouth of the gorge, poses significant disaster risk. Landslides, rockfalls, and debris flows of branches, as the three main geohazards that develop in the valley, transform into material sources under the influence of precipitation and freeze-thaw cycles, resulting in debris flows in the main channel of the Latudi River valley, forming a cascading disaster that affects local socio-economic development. Such geohazards that develop in alpine gorges exhibit characteristics of cascading disasters, high concealment, and remote generation. Early identification through remote sensing can assist in promptly understanding the development of disasters.

As the current main method for early identification of geohazards and cascading geohazards [8], remote sensing has improved the efficiency and accuracy of field surveys. Remote sensing platforms mainly include satellites, unmanned aerial vehicles (UAVs), and aviation aircraft, while remote sensing sensors mainly include synthetic aperture radar (SAR), Light Detection and Ranging (LiDAR) sensors, and optical sensors [8,9]. Optical remote sensing is the earliest remote sensing means used in geohazard surveys. Currently, an increasing number of optical sensors are being mounted on UAVs to meet the demand for convenient and efficient fine-scale geohazard photogrammetry. Both manual visual interpretation and advanced machine learning intelligent interpretation are mainly based on the shape, intensity, texture, and lines of geohazards in optical images [10]. Time-series interferometric SAR (InSAR) analysis based on SAR sensors, such as Persistent Scatterer InSAR (PS-InSAR) and Small Baseline Subset InSAR (SBAS-InSAR), etc., which are capable of detecting surface deformation within a certain period of time, has been widely used in ground subsidence monitoring [11,12,13], earthquake and volcanic activity identification [14,15], and slope-type geohazard identification [16,17]. However, the problem of decoherence and geometric distortion still exists in time-series InSAR in complex terrain areas such as densely vegetated alpine valleys, which remains to be solved. Since the beginning of the 21st century, LiDAR has become an emerging means of geohazard investigation. In addition to traditional terrain mapping functions [18], LiDAR can also identify highly concealed geohazards under vegetation cover. LiDAR sensors are mainly mounted on UAVs or aviation aircraft to scan the ground and obtain point cloud datasets [19,20], while a few sensors are mounted on ground platforms for local area scanning and monitoring [21,22,23]. In order to visualize geohazards, high-resolution Digital Elevation Models (DEMs) extracted by LiDAR can be further converted into hillshade maps and Red Relief Image Maps (RRIMs) [19,24,25], and then the geohazards can be interpreted based on the topographic and geomorphological features represented on the maps.

Compared to using a single remote sensing technology, the simultaneous use of multi-source remote sensing technologies can better delineate the location, extent, and severity of geohazards [10,26]. Many scholars have also adopted various remote sensing technologies for comprehensive identification and continuous monitoring of geohazards, combined with equipment such as GNSS receivers [27,28], terrestrial laser scanners (TLSs) [29,30], or crack monitors [31].

However, currently, the application of multiple remote sensing technologies for geohazard identification is mostly concentrated in plain or hilly areas, targeting individual small regions such as mines [32], slope units [29], reservoirs [33], etc. In high-altitude mountainous areas with steep terrain (above 2000 m in altitude with a vertical drop of more than 2000 m), especially in alpine gorges with dense vegetation, there is insufficient research on the early identification of high-altitude geohazards and associated cascading geohazards utilizing multi-source remote sensing technology. Therefore, this study, taking the Latudi River valley as a case, simultaneously utilizes three methods—SBAS-InSAR, UAV LiDAR, and photogrammetry—to comprehensively identify the highly concealed landslides, rockfalls, and debris flows in the valley. Additionally, this study summarizes the chain of relationships between these hazards. Moreover, it analyzes the impact of rainfall on surface deformation and compares the filtering parameters of point clouds obtained by LiDAR. This study can serve as a reference for the identification of cascading hazards in densely vegetated alpine valley regions and offer guidance to government agencies in formulating strategies for reducing the risk of disasters.

## 2. Materials and Methods

### 2.1. Study Area

**Figure 1:**
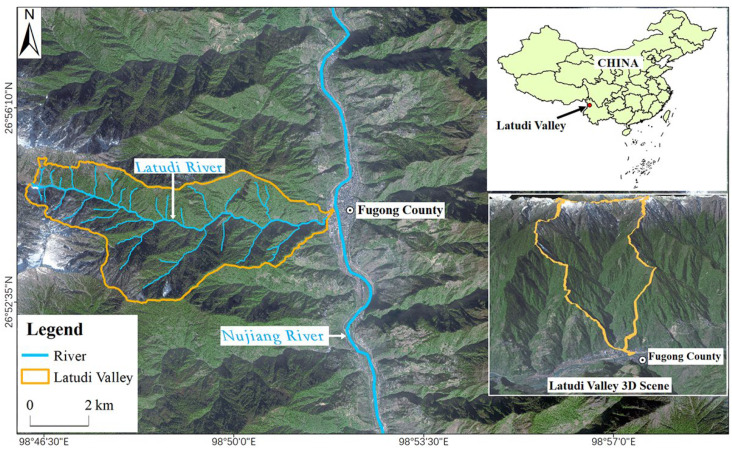
Overview of the geographical location of the Latudi River.

The Latudi River is located in Fugong County, Yunnan Province, southwestern China, within the Hengduan Mountains, which form the eastern segment of the Himalayas. As a primary tributary of the Nujiang River, the valley covers an area of 38.65 km^2^, with a main channel of 13.53 km and a vertical drop of 2631 m. The valley is deeply incised and narrow, with steep terrain, and is nearly upright in parts. The upstream area has sparse vegetation and snow cover, while the middle reaches have dense vegetation, and the downstream area has exposed bedrock, with some areas being cultivated land and villages (Figure 1). The valley has a subtropical mountainous monsoon climate and complex terrain. The climate varies along the river: it is hot along the downstream area, warm along the midstream area, and cold along the upstream area, showing vertical characteristics. The annual precipitation in the area is above 2000 mm.

**Figure 2:**
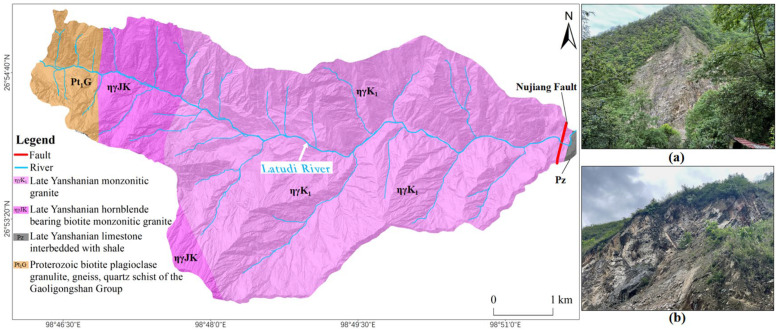
Geological overview of the Latudi River valley. (**a**) The bedrock along the valley bottom is exposed; (**b**) development of fissures in the rock formations.

In terms of geological conditions, the Latudi River valley is influenced by the superimposition of tectonic movements and magma intrusions at different periods, resulting in numerous outcrops of rock formations. The most extensive outcrop is the Late Yanshanian monzonitic granite [34]. The main fault in the valley is the Nujiang Great Fault near the gorge mouth. This fault is located in the collision and compression zone between the Indian Ocean Plate and the Eurasian Plate, running along the Nujiang River’s west bank and roughly longitudinally through the valley. Additionally, according to field surveys, the bedrock along the valley bottom is exposed, with developed fissures (Figure 2).

**Figure 3:**
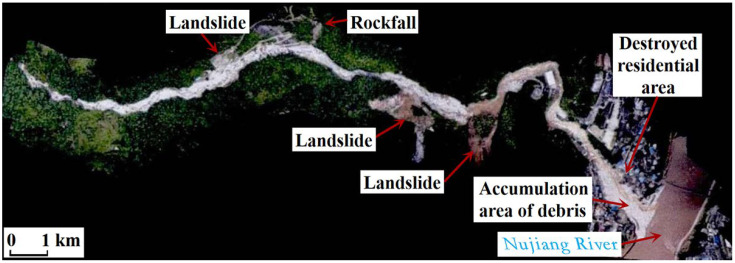
Aerial photo of the lower reaches of the Latudi River in May 2014. Photo from Yunnan Bureau of Surveying, Mapping and Geoinformation.

In the Latudi River valley, after being supplied with material from landslides, rockfalls, and debris flows of branches on both sides of the slope, debris flows occur in the main channel of the Latudi River, forming a disaster chain. When the debris flows rush out of the gorge mouth, they damage buildings and infrastructure, threatening the safety of people’s lives and property. In the event of a large-scale debris flow, there is also a risk of blocking the Nujiang River. So far, multiple disasters have occurred in this valley, with the largest one happening in 2014 (Figure 3). From 2 May to 10 May 2014, continuous rainfall occurred in the valley. On 10 May, between 7:00 a.m. and 11:00 a.m., a catastrophic debris flow broke out in the Latudi River in an intermittent manner, destroying 14 houses, 2 bridges, 2 factories, 1250 m of road, and 325 m of flood embankments.

### 2.2. Data

#### 2.2.1. UAV LiDAR and Photogrammetry Data

The UAV platform that collects LiDAR point clouds and optical images is the Feima D20. As a highly integrated and high-performance UAV, the Feima D20 can withstand winds up to level 6 and has a maximum endurance of 80 min. Additionally, it uses dual differential antennas to provide anti-interference capabilities under complex conditions to ensure flight safety. The LiDAR sensor is mounted on the UAV and includes a laser scanner, imaging device, positioning device, and navigation system. The LiDAR sensor used for point cloud collection is the Feima LiDAR20 (Table 1), with data collected in December 2023, totaling 12 flights, and approximately 230 million points collected. The average point density of the point cloud is 69 points/m^2^. Referring to the “Technical Specifications for Airborne LiDAR Data Acquisition” issued by the China National Administration of Surveying, Mapping, and Geoinformation, and several studies on point cloud density [24,35,36], multiple density calculations were conducted. The density of ground points in the study area reached 21 points/m^2^, meeting the accuracy requirements for producing a DEM with a resolution of 0.5 m. Additionally, the photogrammetric sensor carried on the UAV for this study is Feima D-OP3000 sensor (Table 1). After processing the photogrammetry data, an orthoimage of the study area with a resolution of 0.1 m and cloud cover of less than 5% was obtained.

#### 2.2.2. Sentinel-1A Data and Auxiliary Data

The SAR data used for time-series InSAR are the Sentinel-1A data (Table 1). The Sentinel-1A satellite is a radar satellite launched by the European Space Agency (ESA) in 2014. It is equipped with a C-band SAR sensor with a wavelength of 5.56 cm, which can provide all-weather earth observation imaging. This study collected Sentinel-1A’s first-level product Single Look Complex (SLC) radar images, with a time interval of four years from December 2019 to December 2023. Considering the efficiency of image processing and the coherence of processing results, 59 scenes of descending orbit images with VV polarization, IW imaging mode, and a time interval of 24 days were collected for SBAS-InSAR processing.

In this study, GACOS atmospheric products and Precise Orbit Data (POD) of the Sentinel-1A satellite were utilized as auxiliary data for error correction. Atmospheric effects and orbit deviations are among the main sources of error for radar waves collected by SAR sensors. GACOS atmospheric products can effectively reduce the impact of the troposphere on radar waves [37,38]. The POD of the Sentinel-1A satellite regularly released by the ESA can effectively reduce the systematic errors caused by orbit deviations. Additionally, the DEM of the study area extracted from LiDAR data was used for time-series InSAR to remove the terrain phases.

In order to analyze the impact of rainfall on surface deformation, monthly precipitation data for the Latudi River valley from December 2019 to December 2023 were collected from the Yunnan Provincial Meteorological Agency in China. The meteorological station recording the rainfall data is located in Fugong County, at the mouth of the Latudi River valley.

### 2.3. Method

This study, based on multi-source remote sensing data, identifies landslides, rockfalls, and debris flows that constitute the cascading geohazards in the Latudi River valley. First, the SBAS-InSAR technology is utilized to obtain surface deformation information on the study area, combined with optical imagery for a comprehensive survey of geohazards. Due to the steep terrain and dense vegetation in the study area, InSAR suffers from inevitable coherence loss. Therefore, the UAV LiDAR is further utilized for detailed identification of geohazards. The point cloud dataset obtained by LiDAR is processed through noise reduction and filtering, and then interpolated to obtain the DEM and hillshade map of the study area. Based on microtopographic features such as cracks, back walls, deposits, and hummocks in the hillshade map, highly concealed geohazards under the vegetation can be identified.

#### 2.3.1. Processing of SAR Data with SBAS-InSAR

The time-series InSAR method utilized in this study to process SAR data is the SBAS-InSAR method. This algorithm improves the decoherence phenomenon caused by long spatiotemporal baselines and exhibits superiority in identifying surface deformation in natural areas [39]. SBAS-InSAR performs interferometry on multiple Sentinel-1A SAR images collected over different times using spatiotemporal baseline connections. Deformation inversion is then conducted using a combination of the least squares method and singular value decomposition. The specific principles and processes are as follows:

Assuming N + 1 SAR images of different time periods covering a certain area are acquired, one image is selected as the master image from all images, and the remaining N images are registered to this master image. Based on the selected thresholds for spatial and temporal baselines, all images are freely connected and paired, resulting in M image pairs as Equation (1):

(1) $$\frac{N+1}{2}\ll M\ll N(\frac{N+1}{2})$$

Interference processing is performed on each set of SAR images to obtain M interference patterns. Assuming time t_A_ is the starting time, the SAR image at time t_B_ is paired and interfered with the SAR image at time t_A_, and the i-th interference pattern (1 ≤ i ≤ M) is obtained. The interference phase can be expressed as Equations (2) and (3):

(2) $$\Delta \varphi_{i}=\varphi t_{B}-\varphi t_{A}\approx \Delta \varphi_{\mathrm{def}}+\Delta \varphi_{\mathrm{topo}}+\Delta_{\mathrm{atm}}+\Delta_{\mathrm{noise}}$$

(3) $$\varphi_{\mathrm{def}}=\frac{4\pi }{\lambda }(d_{B}-d_{A})$$

where φt_B_ and φt_A_ represent the phases of the images at times t_B_ and t_A_, respectively. Δφ_def_ represents the deformation phase, Δφ_topo_ represents the topographic phase, Δ_atm_ represents the phase caused by atmospheric delay, and Δ_noise_ represents the phase caused by noise. d_B_ and d_A_ represent the deformation values of the radar line of sight (LOS) at times t_B_ and t_A_, respectively, relative to the start of the entire time series. Atmospheric delay errors, orbit errors, and topographic phases can be removed based on GACOS products, POD, and DEM of the study area. Then, assuming that the surface deformation within the time interval of each pair of interference pairs satisfies linear changes, the deformation phase can be expressed as Equations (4) and (5):

φ_def_ = Av (4)

v = (A^T^A)^−1^A^T^φ_def_ (5)

where φ_def_ represents the deformation phase, A is an m × n coefficient matrix, and v is the phase deformation rate to be solved for each time period. When the matrix A is full rank, a unique deformation rate can be obtained using the least squares method. When the matrix A is not full rank, the generalized inverse matrix of matrix A can be obtained using SVD, and then the deformation rate can be calculated. After obtaining the phase deformation rate, the surface deformation rate can be further obtained.

**Figure 4:**
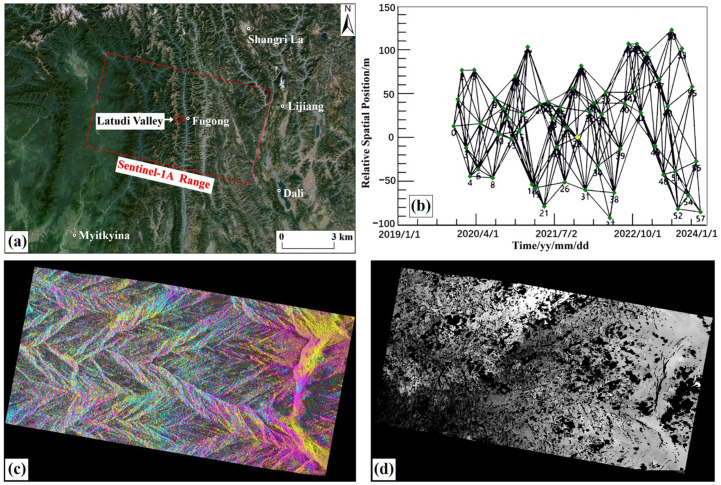
SBAS-InSAR main processing flow. (**a**) Coverage area of each acquired Sentinel-1A image; (**b**) SBAS-InSAR spatial baseline map; (**c**) phase interference map; (**d**) phase unwrapping map.

In this study, during SBAS-InSAR processing, the time baseline threshold was set to 180 d, and the spatial baseline threshold was set to 360 m. After inspection, 252 interference image pairs with good effects were retained. After baseline connection and pairing, image pair interference, phase unwrapping, and deformation inversion (Figure 4), the deformation information of the Latudi River valley was obtained.

#### 2.3.2. Processing of UAV LiDAR and Photogrammetry Data

Time-series InSAR methods such as SBAS-InSAR have qualitative and quantitative measurement capabilities and can provide information for geohazard identification, monitoring, and early warning processes. However, for densely vegetated alpine gorges like the Latudi River valley, InSAR suffers from decoherence and geometric errors. A single remote sensing method cannot accurately and efficiently identify highly concealed geological hazards. Further detailed survey is required in combination with the LiDAR.

**Figure 5:**
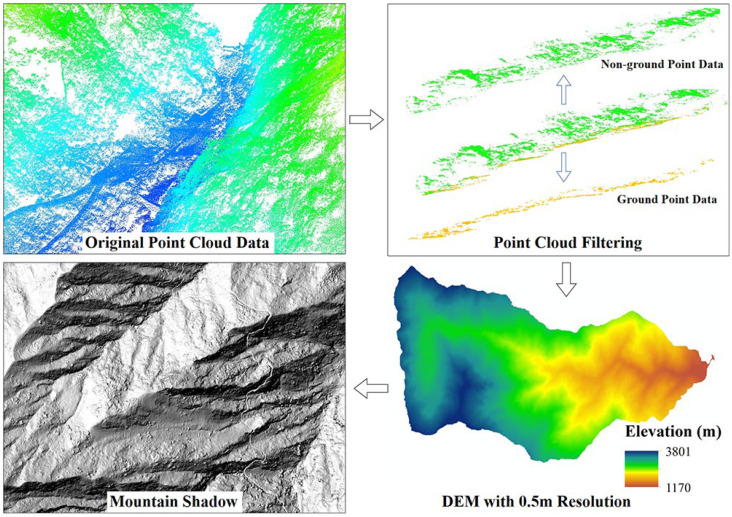
Processing process of point cloud data.

The LiDAR sensor scans the ground surface to obtain a point cloud dataset with coordinate information. After filtering out non-ground points such as vegetation, the ground points are retained. The ground point cloud is then rasterized, and interpolation is performed on the ground point cloud within each grid cell to obtain the surface elevation for each grid, generating a DEM. The DEM is further used to generate a hillshade map, which can achieve the visualization of microtopographic features of geohazards beneath the vegetation cover (Figure 5).

**Figure 6:**
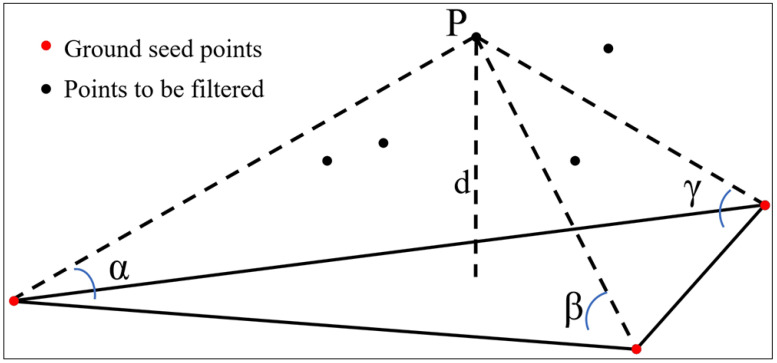
Diagram of IPTD filtering principle.

The key step in point cloud processing is filtering. Zhao et al. [40] proposed the IPTD filtering algorithm in 2016, which combines the traditional progressive triangulated irregular network (TIN) densification (PTD) algorithm [41] and the morphological filtering algorithm [42,43]. The principle is to first select low elevation points as initial ground seed points P and construct an initial TIN according to the morphological filtering. Then, based on the angle (α, β, γ) threshold and distance (d) threshold between the points to be classified and the triangles, more points are gradually classified as ground points or non-ground points (Figure 6). Compared with the traditional PTD algorithm or other filtering methods, the IPTD comprehensively selects ground seed points utilizing morphological methods, resulting in better filtering effects in areas with complex topography such as steep slopes, cliffs, and ground cracks, and preserving finer terrain details [44,45]. Utilizing the IPTD algorithm to filter ground points mainly includes the following four steps: (1) utilizing morphological methods to obtain potential ground seed points; (2) filtering potential ground points to obtain accurate ground seed points; (3) setting parameters for PTD filtering, including angle thresholds (α, β, γ), height difference thresholds (d), iteration times, maximum slope of the study area, etc. [45,46]; (4) establishing and iteratively refining TIN to filter out all ground points. The interpolation of ground points resulted in a 0.5 m resolution DEM of the Latudi River valley.

In this study, the optical sensor, like the LiDAR sensor, was carried on the UAV to capture photogrammetry data. After geometric correction, image fusion, and stitching, an orthoimage of the study area was obtained. Due to the dense vegetation and complex terrain of the study area, the identification of geohazards was primarily conducted using the SBAS-InSAR and the LiDAR. Optical images served as auxiliary data, mainly aiding in the identification of geohazards through visual interpretation based on the shape, tone, texture, and other features displayed in the image.

## 3. Results

### 3.1. Identification Results of SBAS-InSAR

**Figure 7:**
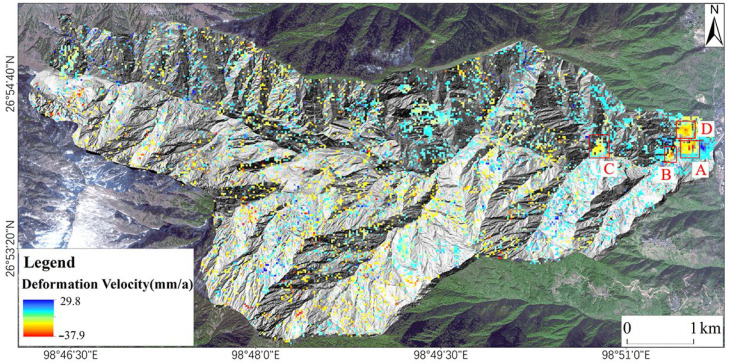
Deformation velocity of Latudi River valley based on SBAS-InSAR.

After processing the Sentinel-1A images of the Latudi River valley with SBAS-InSAR, the LOS surface deformation was obtained. The average annual deformation rate is between −37.9 and 29.8 mm/a (Figure 7). The study area is located in densely vegetated alpine gorges, where SAR images exhibit geometric distortions (overlap, shadows) and reduced coherence, leading to scattered invalid deformation points in most areas. However, there are residual and slope sediments covering the area near the mouth of the valley. Under the combined effects of rainfall, disturbance from human activities, and water erosion, the deformation is obvious. A total of four areas with obvious deformation were identified utilizing SBAS-InSAR, which are marked as areas A, B, C, and D, respectively, in Figure 7.

**Figure 8:**
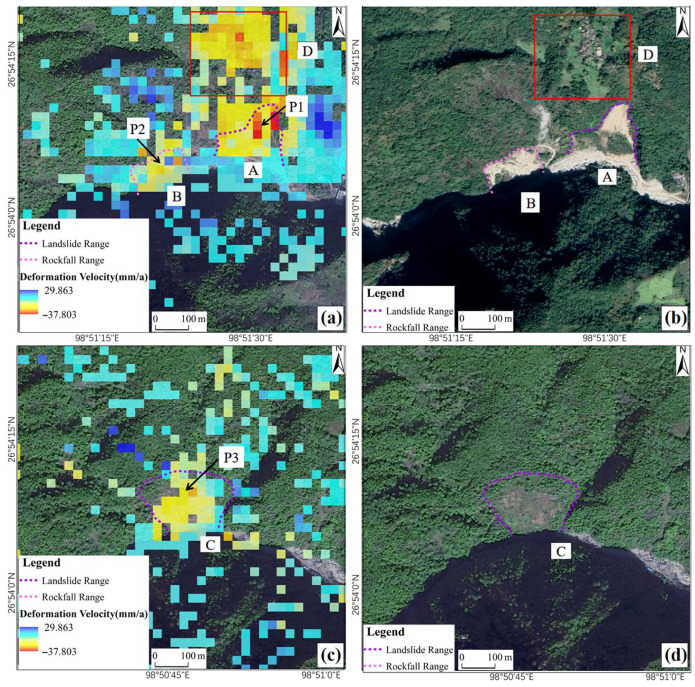
Identified geohazard areas A, B, and C. (**a**) Deformation rate map of areas A, B, and D; (**b**) UAV image of areas A, B, and D; (**c**) deformation rate map of area C; (**d**) UAV image of area C.

Through field verification and comprehensive interpretation of UAV orthoimages, it was determined that areas A and C are landslides, area B is a rockfall, and area D is a village and farmland (Figure 8). The sliding length of landslide A is about 274 m, the width is about 177 m, the area is about 37,087 m^2^, and the average subsidence rate is about 22 mm/a. The sliding length of landslide C is about 191 m, the width is about 216 m, the area is about 36,188 m^2^, and the average subsidence rate is about 11 mm/a. The length of rockfall B is about 110 m, the width is about 183 m, the area is about 10,606 m^2^, and the average subsidence rate is about 13 mm/a. Area D is the village and farmland subsidence area, with an average subsidence rate of about 19 mm/a.

Combined with a field survey, an analysis of the cause of deformation in the four areas was conducted. The front edges of geohazards A, B, and C were all eroded by the Latudi River water or there were slope cutting projects such as road construction projects, which destroyed the shear resistance of the slope, thus reducing the stability of the slope, and the rear edge was under traction. Cracks gradually develop, and the landslides or rockfall bodies become loose, deformed, and unstable. With the occurrence of landslide A and rockfall B, the villages and farmland in area D on the top of the slope were also affected by rainfall. The surface water content became saturated and overall subsidence occurred.

### 3.2. Identification Results of UAV LiDAR

**Figure 9:**
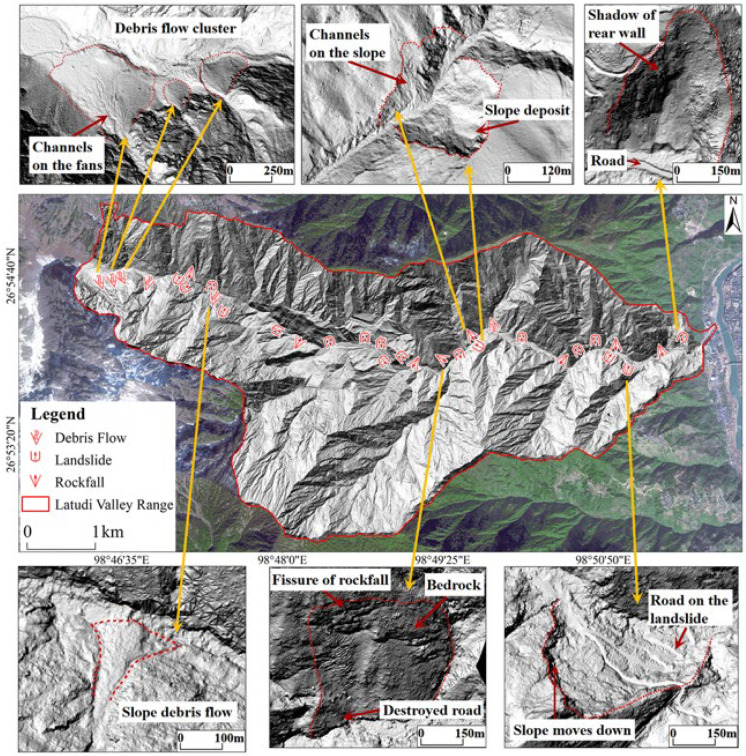
Identification results and examples of geohazards in the Latudi River valley.

Based on airborne LiDAR data, a total of 32 geohazards were identified in the Latudi River valley, including 18 landslides, 8 rockfalls, and 6 debris flows in tributary gullies (Figure 9). Geohazards are evenly distributed on the northern and southern slopes of the valley, and their spatial distribution from the valley mouth to the valley end is also relatively even. The geohazard locations are all near the banks of the river, indicating that hydrodynamic action is a significant controlling factor of hazards. The front edges of landslides and rockfalls are continuously eroded by flowing water, leading to decreased slope stability. During the rainy season, as rainfall increases, the weight of the slope increases while shear resistance decreases. Consequently, the front edge of landslides is continuously eroded, and cracks form at the rear edge under the influence of flowing water, leading to deformation. Among the six debris flows in tributary gullies, four are located at the valley end, possibly due to the higher altitude, sparse vegetation, and ice and snow cover in the debris flow provenance, which can provide greater force and more abundant loose source material when ice and snow melt.

**Figure 10:**
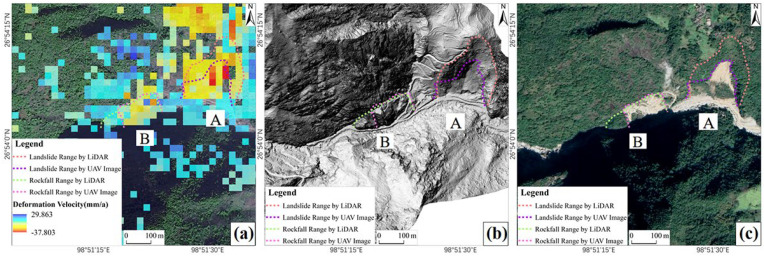
Comparison of identification results from different remote sensing methods. (**a**) Identification results from SBAS-InSAR. (**b**) Identification results from LiDAR. (**c**) Identification results from UAV optical image.

In the comparison between the identification results of SBAS-InSAR and LiDAR, it was found that SBAS-InSAR and UAV optical images can identify the rough deformation range, while the more detailed range of geohazards needs to be identified with LiDAR. For example, for landslide A covered with dense vegetation, LiDAR can interpret a more accurate range of the hazard body beneath the vegetation (Figure 10).

#### 3.2.1. Identification Results for Landslides

**Figure 11:**
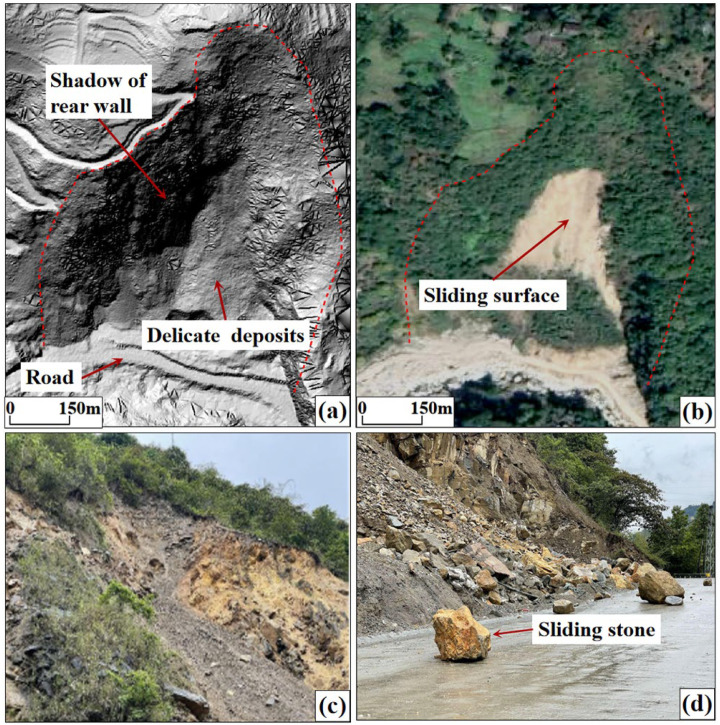
Landslide features. (**a**) Hillshade map; (**b**) UAV image; (**c**) field survey photo—landslide rear wall; (**d**) field survey photo—landslide front edge.

Landslides mainly develop along the Latudi River, exhibiting various shapes such as semicircular niches by concave slopes, tongues, and bowl shapes. On the hillshade map [46], shadows formed by the landslide mass can be observed on the rear wall. The texture of the landslide bodies is fine, appearing brighter in orthoimages. Some landslides exhibit features such as the main scarp, boundary, accumulation area, and tension cracks. In the orthoimage, the vegetation coverage within the landslide is low and mostly consists of weeds and short shrubs (Figure 11).

#### 3.2.2. Identification Results for Rockfalls

**Figure 12:**
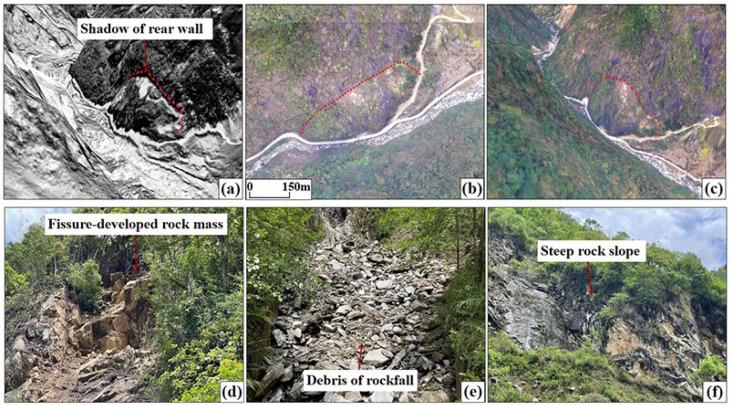
Rockfall features. (**a**) Three-dimensional hillshade map; (**b**) UAV image; (**c**) three-dimensional image; (**d**) field survey photo—landslide site; (**e**) field survey photo—collapsed debris; (**f**) field survey photo—rockfall surface.

The rockfalls mainly occur on steep slopes with free surfaces above 60 degrees along the Latudi River. The rock mass is fragmented, and tensile cracks and fractured rock structures are visible on the back wall. The collapsed debris accumulates at the foot of the slope, forming an inverted cone shape. The shape and particle size can be distinguished from the high-precision hillshade map (Figure 12).

#### 3.2.3. Identification Results for Debris Flows

**Figure 13:**
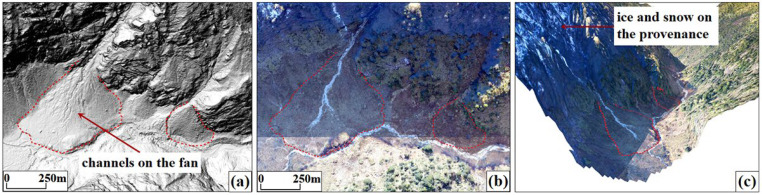
Debris flow features. (**a**) Hillshade map; (**b**) UAV image; (**c**) 3D scene.

Debris flows develop in the tributaries of the Latudi River with large slope ratios. They have complete source areas, circulation areas, and accumulation areas. A typical identification feature is the accumulation fans, with fine-grained textures and evidence of water erosion on their surfaces. The middle and upper reaches of the tributaries have a high altitude, and the provenance is covered with ice and snow. When the ice and snow melt, they can provide power and material sources (Figure 13).

## 4. Discussion

### 4.1. The Impact of Rainfall on Deformation

**Figure 14:**
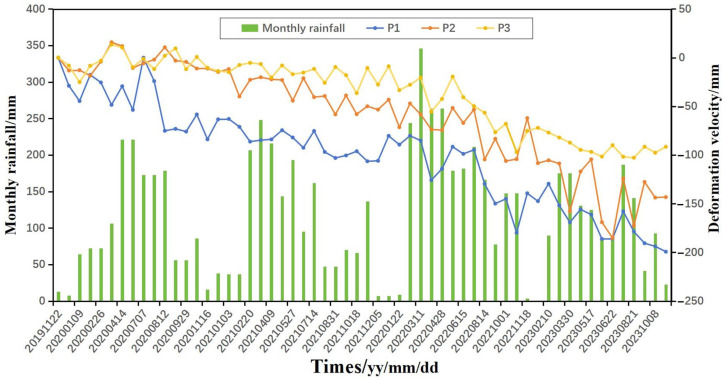
The relationship between deformation points P1, P2, and P3 and monthly average rainfall.

In order to explore the impact of rainfall on surface deformation in the Latudi River valley, a time-series analysis was conducted on the monthly deformation variables at points P1, P2, and P3 located at the rear walls of geohazards A, B, and C, respectively (Figure 14), coupled with monthly average rainfall data. The maximum subsidence values of P1, P2, and P3 are approximately 200 mm, 145 mm, and 85 mm, respectively. Due to the erosion by flowing water at the slope’s forefront, all three points show an accelerating downward trend. P1 and P2, being closer to the gorge mouth, experience stronger hydrodynamic forces, resulting in more severe erosion. Additionally, human engineering activities such as road construction have reduced the stability of the slope, leading to greater subsidence compared to P3. In 2022, when rainfall was higher, the subsidence values at all three points were also higher compared to the other three years. Additionally, during the periods of highest rainfall in 2022, the corresponding deformation values did not sharply decrease simultaneously, indicating that slope deformation lagged behind intense rainfall events. This is because after rainfall infiltration, the increase in weight of the geohazard mass requires cumulative time; subsequently, the hazard bodies slide and collapse along weakened structural planes under the influence of gravity [47]. In terms of climate, due to the dual influence of the Indian Ocean monsoon and the Pacific monsoon, the Latudi River valley has two rainy seasons, from February to April and from June to September. Between these, February to April is the main rainy season, and about 65% of heavy rains occur during this period. Overall, the three points experienced greater subsidence from February to April.

### 4.2. Analysis of Point Cloud Filtering Algorithm

**Figure 15:**
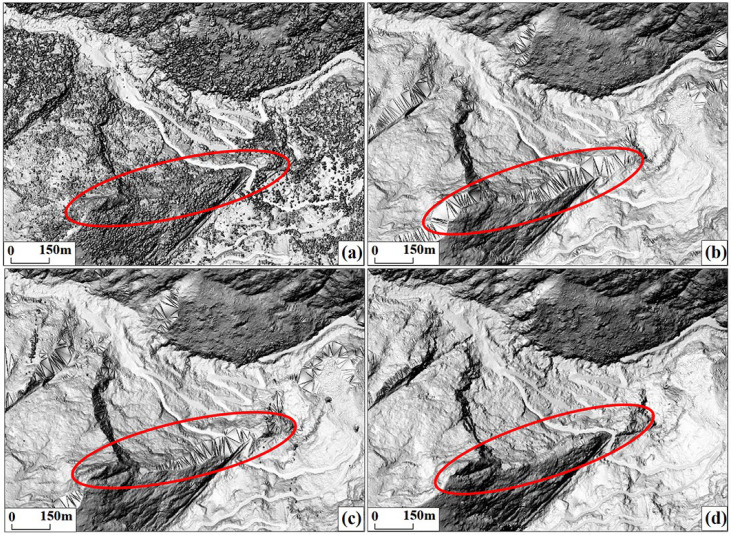
Based on the IPTD algorithm, the filtering effects of steep terrain obtained with different angle and distance thresholds are displayed. (**a**) Angle/distance: 55°/1.5 m; (**b**) angle/distance: 30°/0.8 m; (**c**) angle/distance: 30°/1.6 m; (**d**) angle/distance: 30°/1.6 m and 45°/1.5 m.

When utilizing IPTD for point cloud filtering, the main parameters are the angles α, β, and γ between the point to be classified and the three vertices of the triangle, as well as the vertical distance d from the point to be classified to the triangle. In this study, in order to select the most suitable parameters for the Latudi River valley, the angle and distance parameters were set to the following nine groups: 15°/0.8 m; 15°/0.2 m; 30°/0.8 m; 30°/1.5 m; 30°/1.6 m; 45°/1.5 m; 50°/1.5 m; 55°/1.5 m; and 45°/1.5 m. After multiple tests, the parameters finally adopted were 30°/1.6 m and 45°/1.5 m. When using these two sets of parameters, the ground points on the mountain tops and steep slopes can be retained best, and fractures with large terrain undulations can be processed. The specific setting method is as follows: First, the angle and distance thresholds are set to 30°/1.6 m to extract ground points. Second, for the remaining non-ground points selected in the first step, the angle and distance thresholds are set to 45°/1.5 m for the second extraction of ground points. The point clouds extracted twice are merged as the ground points of the study area, and further interpolated into a DEM with a resolution of 0.5 m (Figure 15).

### 4.3. Cascading Relationship Analysis of Geohazards

**Figure 16:**
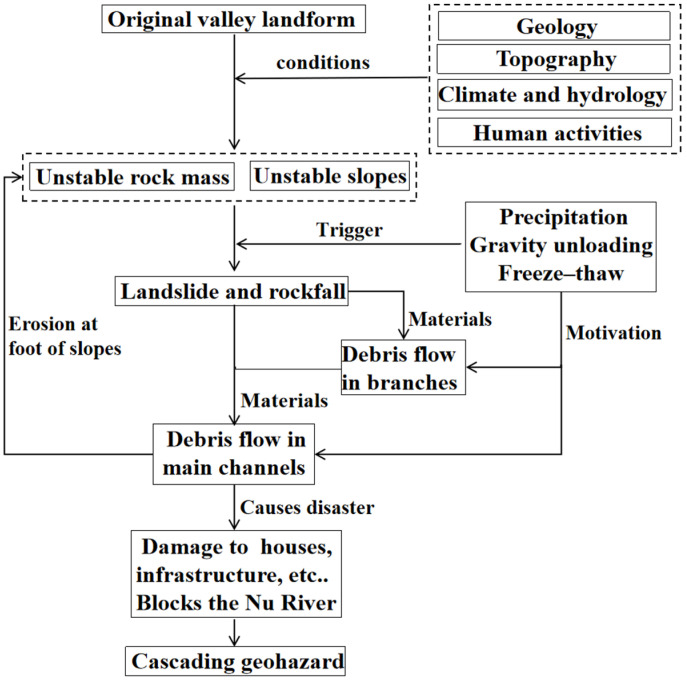
Cascading relationship of geohazards in the Latudi River valley.

The geohazards that have developed in the Latudi River valley mainly include landslides, rockfalls, and debris flows, which are interrelated, are conditional on each other, and appear sequentially. Based on the historical occurrences of disasters in this valley, particularly the large-scale debris flow disaster on 10 May 2014, the cascading relationship of geohazards in the Latudi River valley is summarized in Figure 16. Under the influence of four factors including geological conditions, topography, climate, and hydrology, and human activities, the original slopes gradually evolve into unstable slopes and dangerous rock masses. Under the triggering of factors such as precipitation and freeze–thaw action, landslides and rockfalls occur. As the source material, debris from landslides and rockfalls, driven by rainfall, triggers debris flows in the tributaries and the main channel of the Latudi River. At the same time, debris flows from the tributaries rushing into the main channel can also provide source materials for the main channel debris flows [48]. During the high-speed movement of debris flows, erosion of the slopes on both sides further reduces slope stability [49]. After the debris flow rushes out of the gorge mouth, it damages or buries houses and infrastructure. If a large-scale debris flow breaks out, there is also a risk of blocking the Nujiang River at the gorge mouth. Through the above cascading relationship, the cascading geohazards in the Latudi River valley eventually result in disaster.

## 5. Conclusions

This study takes the Latudi River valley located in the alpine gorge as the research area, comprehensively combining three remote sensing methods—UAV LiDAR, time-series InSAR, and UAV photogrammetry—to identify landslides, rockfalls, and debris flows within the valley.

Within the entire 38 km^2^ Latudi River valley, a total of 18 landslides, 8 rockfalls, and 6 debris flows in tributary gullies were identified. These three types of geohazards have a cascading relationship and are distributed along the banks of the Latudi River, significantly influenced by its hydrodynamic forces. Additionally, the development of geohazards within the valley is significantly influenced by climate and precipitation. Surface deformation varies with rainfall, with the highest surface deformation observed in 2022, which had the highest rainfall. The use of LiDAR greatly improved the identification of geohazards, but the choice of point cloud filtering parameters had a significant impact on the accuracy of the extracted DEM. After experimenting and comparing different parameters for the IPTD algorithm, it was found that the best results were achieved by filtering the point cloud twice using two sets of parameters. Continuous rainfall and erosion of the side slopes by flowing water are the main factors contributing to the formation of debris flow disasters in the main channel of the Latudi River. This study can provide a reference for government departments to take preventive measures, such as constructing retaining walls at the forefront of the slopes and installing water level monitors downstream in the valley.

This study shows that in alpine gorges with dense vegetation and steep terrain, such as the Latudi River valley, the three remote sensing methods used can mutually confirm and complement each other in the identification of geohazards and their associated hazard cascades, as summarized in Table 2.

## Figures and Tables

**Table 1:** Introduction of data sources.

Data Source	UAV LiDAR	UAV Photogrammetry	Sentinel-1A SAR
Platform and sensor parameters	LiDAR sensor: D-LIDAR2200 Manufacturer: Shenzhen Feima Robotics Co., Ltd., Shenzhen, China Flight height: 50–200 m Point accuracy: ±5 cm Number of echoes: 3 times Horizontal field of view: 61° Vertical field of view: 43°	Photogrammetry sensor: D-OP3000 Manufacturer: Shenzhen Feima Robotics Co., Ltd., Shenzhen, China Camera: SONY a6000 number of pixels: 24.3 million Down view focal length: 25 mm Tilt focal length: 35 mm	Satellite: Sentinel-1A Affiliated organization: European Space Agency (ESA) Orbit: Near-polar synchronous orbit Orbital altitude: 693 km Band: C-band Wavelength: 5.6 cm Return period: 12 d Selected imaging mode: IW
Processing method	Improved progressive triangulated irregular network densification (IPTD) filtering	Comprehensive interpretation	SBAS-InSAR
Processing result	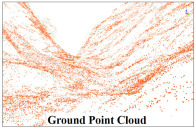	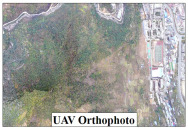	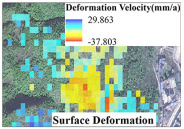
Accuracy	0.5 m resolution	0.1 m resolution	Millimeter-scale

**Table 2:** Comparison of different remote sensing sensors used to identify geohazards in alpine gorge areas.

Identification Methods	Products	Efficiency	Accessibility	Features
Manual survey	Field findings	Low	Difficult	Difficult to survey
UAV photogrammetry	Orthoimages	Medium	Easy	Disturbed by dense vegetation
Time-series InSAR	Time series of surface deformation	High	Easy	Geometric distortion; disturbed by vegetation
UAV LiDAR	High-resolution DEMs or hillshade maps	High	Easy	Geohazard micro-landforms identified under vegetation

## Data Availability

The Sentinel-1 datasets can be acquired freely from the Copernicus and ESA: https://search.asf.alaska.edu/#/, accessed on 5 January 2024.
